# Prolonged Antibiotic Use in a Preclinical Model of Gulf War Chronic Multisymptom-Illness Causes Renal Fibrosis-like Pathology via Increased micro-RNA 21-Induced PTEN Inhibition That Is Correlated with Low Host *Lachnospiraceae* Abundance

**DOI:** 10.3390/cells13010056

**Published:** 2023-12-27

**Authors:** Ayushi Trivedi, Dipro Bose, Punnag Saha, Subhajit Roy, Madhura More, Jonathan Skupsky, Nancy G. Klimas, Saurabh Chatterjee

**Affiliations:** 1 Environmental Health and Disease Laboratory, Department of Environmental and Occupational Health, Program in Public Health, Susan and Henry Samueli College of Health Sciences, University of California, Irvine, CA 92697, USA; aktrived@uci.edu (A.T.); diprob@uci.edu (D.B.); punnags@uci.edu (P.S.); subhajr@uci.edu (S.R.); mpmore@uci.edu (M.M.); 2 Long Beach VA Medical Center, Long Beach, CA 90822, USA; jonathan.skupsky@va.gov; 3 Institute for Neuro-Immune Medicine, College of Osteopathic Medicine, Nova Southeastern University, Fort Lauderdale, FL 33328, USA; nklimas@nova.edu; 4 Division of Infectious Diseases, Department of Medicine, School of Medicine, University of California, Irvine, CA 92697, USA

**Keywords:** Prolong antibiotics, Gulf War Illness, TGF-β, miR-21, PTEN, *Lachnospiraceae* spp., renal fibrosis

## Abstract

Gulf War (GW) veterans show gastrointestinal disturbances and gut dysbiosis. Prolonged antibiotic treatments commonly employed in veterans, especially the use of fluoroquinolones and aminoglycosides, have also been associated with dysbiosis. This study investigates the effect of prolonged antibiotic exposure on risks of adverse renal pathology and its association with gut bacterial species abundance in underlying GWI and aims to uncover the molecular mechanisms leading to possible renal dysfunction with aging. Using a GWI mouse model, administration of a prolonged antibiotic regimen involving neomycin and enrofloxacin treatment for 5 months showed an exacerbated renal inflammation with increased NF-κB activation and pro-inflammatory cytokines levels. Involvement of the high mobility group 1 (HMGB1)-mediated receptor for advanced glycation end products (RAGE) activation triggered an inflammatory phenotype and increased transforming growth factor-β (TGF-β) production. Mechanistically, TGF-β- induced microRNA-21 upregulation in the renal tissue leads to decreased phosphatase and tensin homolog (PTEN) expression. The above event led to the activation of protein kinase-B (AKT) signaling, resulting in increased fibronectin production and fibrosis-like pathology. Importantly, the increased miR-21 was associated with low levels of *Lachnospiraceae* in the host gut which is also a key to heightened HMGB1-mediated inflammation. Overall, though correlative, the study highlights the complex interplay between GWI, host gut dysbiosis, prolonged antibiotics usage, and renal pathology via miR-21/PTEN/AKT signaling.

## 1. Introduction

Gulf War Illness (GWI), a chronic and complex medical condition affecting Persian Gulf War veterans, presents a wide range of symptoms, including gastrointestinal (GI) disturbances, pain, fatigue, and cognitive impairments. Its exact cause remains elusive, but it is believed to result from various factors, including exposure to Pyridostigmine bromide, insecticides, sarin, and psychosocial conditions during the Gulf War [1,2]. A 2012 epidemiological study by L. Steele et al. highlighted the significant role of pesticides and Pyridostigmine bromide exposure as risk factors contributing to GWI development [3]. Our lab was the first to establish the role of gut dysbiosis as a key contributor to GI and neuro-inflammation in preclinical models of GWI. The study showed that gut microbiome dysbiosis results in disruption in tight junction protein levels, particularly Occludin and Claudin-2, thereby compromising the intestinal barrier integrity, which leads to systemic endotoxemia resulting in Toll-like receptor-4 (TLR4) activation-mediated oxidative stress and gastrointestinal and neuroinflammation [4]. Another pilot study conducted in 2019 involving GW veterans who participated in the Boston GWI Consortium study reported that GW veterans with GWI and GI symptoms had a significantly different gut composition when compared with GW veterans with GWI but no GI symptoms or GW controls [5]. Later, in 2021, Bose et al. reported that this gut dysbiosis persisted even after 20 weeks of exposure to GW chemicals, which is roughly equivalent to 15.5 years in GW veterans, leading to compromised intestinal barrier integrity with elevated serum Interleukin-1β (IL-1β) and Interleukin-6 (IL-6) level-mediated hepatic inflammation and neurocognitive deficiencies [6]. Given the enduring presence of gut dysbiosis in Gulf War veterans suffering from GWI and related symptoms, it is plausible that they may have been exposed to a variety of broad-spectrum antibiotics over their lifetime as part of medical interventions aimed at addressing these conditions. Further, an underlying condition of GWI may give rise to heightened risks of renal disease as the veterans age since most of them who served during the 1990–1991 deployment have an average age of 55 years. Interestingly, gut dysbiosis and immunosenescence are linked to chronic kidney disease (CKD), a manifestation of aging-related conditions [7].

To date, we are not aware of any clinical evidence that correlates the exposure to GW chemicals, particularly Pyridostigmine bromide and permethrin with renal pathology. The overt use of antibiotics of the specified classes fluoroquinolones, aminoglycosides, and β-lactams are known to cause renal disease pathologies [8]; however, the intensity of damage in GW veterans using long-term antibiotics could manifest into exacerbated renal pathologies due to the complex nature and ectopic effects of GW chemicals, especially in uninvestigated organs such as kidneys. Hence, we sought to focus on examining the influence of GW chemical exposure, the persistence of host gut dysbiosis, and possible risks of renal disease development.

Renal fibrosis is characterized by increased extracellular matrix protein (ECM) deposition such as fibronectin and collagen, and increased accumulation of myofibroblasts [9]. HMGB1 is expressed by many renal cell types and serves as a damage-associated molecular pattern molecule (DAMP) in the extracellular environment, thereby contributing to pathogenesis in various renal diseases. HMGB1 interacts with its downstream receptors such as Toll-like receptors (TLR2, TLR4, and TLR9), RAGE, and integrin, and functions as a pro-inflammatory factor [10]. Activation of RAGE by extracellular HMGB1 triggers NFκB signaling and ultimately induces an epithelial–mesenchymal transition (EMT)-like phenotype in proximal tubular epithelial cells (PTECs) via TGF-β production [11]. TGF-β functions as a master regulator of renal inflammation and fibrosis by increasing the accumulation of ECM proteins and development of EMT in the tubule-interstitium and glomerular region of the kidneys [12,13,14,15].

MicroRNAs are small, non-coding, endogenous RNAs that play a significant role in cellular proliferation, apoptosis, metabolism, and pathophysiology of diseases by regulating target mRNA transcripts. Aberrant expression of miRNAs such as miR-192, miR-433, miR-26a-5p, miR-21, miR-29, and miR-140-5p have been strongly associated with renal fibrosis [16,17,18,19,20,21]. Upregulation of miR-21 mediated by TGF-β production has been widely reported in kidney diseases such as renal fibrosis, glomerular injury, and CKD [22,23,24]. Additionally, previous research has suggested that miR-21 targets PTEN expression [25], which negatively regulates the AKT signaling pathway, indicating the role of TGF-β-induced miR-21/PTEN/AKT signaling-mediated renal fibrosis pathology [26,27]. 

Therefore, this study aims to decipher the effect of prolonged antibiotic exposure (5 mouse months), especially of classes fluoroquinolone–enrofloxacin and aminoglycoside–neomycin and GW chemical exposure on renal pathology in a persistent GWI mouse model. It also aims to elucidate the underlying molecular mechanisms leading to renal pathology by mainly focusing on TGF-β-mediated activation of miR-21/PTEN/AKT signaling. 

## 2. Materials and Methods

### 2.1. Materials

Chemicals employed in this research, such as Pyridostigmine bromide (PB), Permethrin (Per), enrofloxacin, and neomycin, were procured from Sigma-Aldrich (St. Louis, MO, USA). The antibodies utilized in this study, such as anti-Receptor for Advanced Glycation End Products (RAGE) (sc-365154), anti-Fibronectin (sc-52331), and anti-TGF-β (sc-130348) were obtained from Santa Cruz Biotechnology (Dallas, TX, USA). Anti-HMGB1 (10829-1-AP) and anti-Tumor Necrosis Factor-α (TNF-α) (17590-1-AP) were purchased from Proteintech (Rosemont, IL, USA), while anti-PTEN (ab32199), anti-IL-17A (ab79056), and anti-IL-1β (ab9722) antibodies, along with the Diaminobenzidine (DAB) Substrate Kit (ab64238), were procured from Abcam (Cambridge, MA, USA). Other primary antibodies targeting phosphorylated-AKT [S473] (4060S), total-AKT (9272S), phosphorylated-Nuclear Factor κB p65 [S536] (3033S), total-Nuclear Factor κB p65 (4764S), and species-specific secondary antibodies for Western blotting were sourced from Cell Signaling Technology (Danvers, MA, USA). Species-specific biotinylated antibodies and streptavidin horseradish peroxidase (HRP) Vectastain Elite ABC kit were obtained from Vector Laboratories (Burlingame, CA, USA), and Fluorescence-conjugated (Alexa Fluor) secondary antibodies were purchased from Thermo Fisher Scientific (Grand Island, NY, USA). Unless otherwise indicated, all other chemicals and reagents used in this study were acquired from Sigma Aldrich (St. Louis, MO, USA). For the process of paraffin embedding and sectioning of animal tissues, the Experimental Tissue Resource facility at the University of California, Irvine (Irvine, CA, USA) was utilized.

### 2.2. Animal Model and Care

C57BL/6J adult wild-type male mice, aged 10 weeks, procured from the Jackson Laboratories (Bar Harbor, ME, USA) were utilized in this research. All animal handling procedures strictly adhere to the guidelines established by the National Institutes of Health (NIH) for the care and use of laboratory animals and are in compliance with Institutional Animal Care and Use Committee (IACUC) standards. The University of South Carolina, Columbia, SC, USA, granted approval for these animal protocols. The mice were housed under controlled environmental conditions, maintaining a temperature range of 22–24 °C with a 12 h light/12 h dark cycle. The mice had ad libitum access to both food and water throughout the study. Based on our experimental design, the mice were divided into four groups (n = 6) and were fed a standard CHOW diet. After animal experimentation, all mice were humanely euthanized and vital organs including the kidneys were carefully excised. Specifically, one kidney from each mouse was preserved in 10% neutral buffered formaldehyde for histopathological analysis, while the second kidney was collected and snap frozen in liquid nitrogen and stored long term at −80 °C.

#### Mouse Model of Gulf War Illness (GWI)

Following a week of acclimatization, the mice were randomly divided into four groups and received different treatments. The control group (n = 6) was treated with a vehicle control (0.6% DMSO diluted in PBS). The Gulf War Illness group (GWI, n = 6) received Permethrin (200 mg/Kg, diluted in DMSO and PBS; with a final DMSO concentration of 0.6%) and Pyridostigmine bromide (2 mg/Kg, diluted in PBS) through oral gavage on alternate days for 2 weeks. The GWI+AB group (n = 6) was exposed to Permethrin and Pyridostigmine bromide, as in Group 2, and additionally, they were administered daily oral gavage with antibiotics (1 mg/Kg enrofloxacin in DMSO and 45 mg/Kg neomycin in PBS, with a final DMSO concentration of 0.6%) for a duration of 20 weeks (5 months). The AB-only group (n = 6) received enrofloxacin and neomycin at similar concentrations for the same 20-week duration as Group 3. Prolonged antibiotic treatment was employed to mimic the extended use of antibiotics over a 20-week period, simulating the duration of exposure during the Gulf War and the subsequent period following their return from the war.

### 2.3. Microbiome Analysis

We examined the relative abundance of *Lachnospiraceae* spp. based on the microbiome analysis carried out by CosmosID Inc. (Germantown, MD, USA). A ZymoBIOMICS Miniprep kit was used to isolate and purify the total DNA from the mice fecal pellets that were collected. Qubit dsDNA HS Assay (Thermofisher, Waltham, MA, USA) was used to quantify the DNA. A DNA library was prepared using the Illumina Nextera XT kit with modifications. Whole genome sequencing was performed using the Illumina HiSeq 4000 platform (vendor-optimized protocol followed). For sequencing, the average insert size was 1400 bp and a read length of 2 × 150 bp was used. A fast QC followed by a multi-QC report was generated after obtaining the raw data. In order to check that no abnormalities were present in the read quality, adaptor content, or duplication rates, and to confirm that the threshold for the read depth was met, the multi-QC report was reviewed. To confirm that no barcoding or contamination issues were present, the taxonomic results were reviewed on the CosmosID bioinformatics platform (http://app.cosmosid.com (accessed on 21 September 2023)). Subsequently, a bar graph illustrating the percent relative abundance of *Lachnospiraceae* spp. derived from taxonomic analysis was generated using the collected data.

### 2.4. Quantitative Real-Time Polymerase Chain Reaction (qRT-PCR)

To assess gene expression in kidney tissue samples, we employed a two-step qRT-PCR method. First, total RNA was extracted from the kidney tissues using TRIzol reagent (Invitrogen, Waltham, MA, USA) following the manufacturer’s instructions, and then further purification was carried out using RNeasy mini columns (Qiagen, Valencia, CA, USA). 

The purified RNA was subsequently reverse-transcribed into cDNA using the iScript cDNA synthesis kit purchased from Bio-Rad (Hercules, CA, USA) according to the manufacturer’s protocol. Next, we conducted qRT-PCR using gene-specific primers, SsoAdvanced SYBR Green Supermix (Bio-Rad), and an AriaMx Real-time PCR System (Agilent Technologies, Santa Clara, CA, USA). The Ct values for the target genes were normalized to GAPDH (used as an internal control) within the same sample. The 2^−ΔΔCt^ method was used to calculate the relative fold change in gene expression. Detailed information on the mouse-specific primer sequences employed for real-time PCR can be found in Table 1.

#### miR-21 Quantification and Analysis

Total RNA containing miRNA was extracted from kidney tissue samples using a miRNeasy Tissue/Cells Advanced Micro Kit (Cat# 217684, Qiagen) according to the manufacturer’s protocol. Around 300ng of purified miRNA was converted to cDNA and further analysis of miR-21 expression was conducted using a predesigned species-specific primer for mmu-miR-21-5p (Cat No./ID: YP00204230 (339306)) and a miRCURY LNA™ miRNA PCR Starter Kit Mouse (GeneGlobe ID—YKP-MM). All the reactions were performed in triplicates using the AriaMx Real-time PCR System (Agilent Technologies) and the Ct values from the target miRNA were normalized with miR-103a-3p which served as an endogenous control. 

### 2.5. Immunohistochemical Analysis

For the immunohistochemical analysis, deparaffinization of paraffin-embedded kidney sections was performed according to standard laboratory procedures. Following deparaffinization, antigen epitope retrieval was accomplished using an epitope retrieval solution and a steamer (IHC-World, Woodstock, MD, USA). Subsequently, endogenous peroxidase activity was blocked by incubating the tissues in a 3% H_2_O_2_ solution for 20 min, followed by serum blocking using 5% goat serum for 1 h.

After serum blocking, primary antibodies specific to TNF-α, TGFβ1, fibronectin, and α-SMA were diluted in 1:250 ratios in blocking buffer and applied to the tissue sections. Following the overnight incubation at 4 °C, the tissue sections were subjected to wash with 1x PBS-T (PBS + 0.05% Tween 20) thrice.

Biotinylated secondary antibodies specific to the species’ origin were applied at 1:200 dilutions, followed by incubation with streptavidin conjugated with horseradish peroxidase at 1:500 dilutions. Finally, the chromogenic substrate solution of 3,3-diaminobenzidine (DAB) from Sigma-Aldrich was applied to the sections, and counterstaining was performed using Mayer’s hematoxylin (Sigma-Aldrich). The mounted tissue sections were covered with Mounting media (Abcam, Cambridge, MA, USA). Images were captured using an Olympus BX43 microscope (Olympus, Center Valley, PA, USA) and morphometric data analyses were carried out using CellSens Software V2.2 from Olympus America (Center Valley, PA, USA).

### 2.6. Immunofluorescence Staining

In the immunofluorescence staining procedure, deparaffinization and epitope retrieval steps for paraffin-embedded sections of kidney tissues were performed as previously outlined. Subsequent to the epitope retrieval process, the tissue sections were permeabilized using a solution of PBS-T (PBS + 0.1% Triton X-100) for 1 h. Blocking was accomplished using 5% goat serum, after which the sections were incubated with primary antibodies targeting HMGB1 and RAGE and stored at 4 °C overnight. Species-specific secondary antibodies labeled with Alexa Fluor 488 or 633 (Invitrogen) were utilized and, following the incubation process, tissue sections were washed with 1x PBS-T and were mounted using ProLong Gold antifade reagent with DAPI (Life Technologies, Carlsbad, CA, USA). Images were captured using an Olympus BX43 microscope and CellSens Software V2.2 from Olympus America was used for morphometric analysis.

### 2.7. Western Blot Analysis

Total protein from kidney tissues from mice was extracted using 1XRIPA lysis buffer with a protease and phosphatase inhibitor, and the protein concentration was determined using a BCA assay kit purchased from Thermo Fisher Scientific (Rockford, IL, USA). Around 30 µg of protein from each sample was mixed with 4x NuPAGE™ LDS Sample Buffer (Thermo Fisher Scientific, Rockford, IL, USA) and 10% β-mercaptoethanol. Protein separation was performed via SDS-PAGE on a Novex 4–12% bis-tris gradient gel, and subsequent transfer of protein bands to a nitrocellulose membrane was achieved using the Trans-Blot Turbo transfer system (Bio-rad, Hercules, CA, USA). Following the transfer, the membrane was stained with Ponceau S followed by blocking with 3% bovine serum albumin (BSA) in 1x TBST for 1 h. Primary antibodies like anti-IL-1β, anti-IL-17A, anti-PTEN, anti-phosphorylated-AKT, anti-total-AKT, anti-phosphorylated-p65, anti-total p65, and anti-β-actin were diluted in a 1:1000 ratio in 1% BSA and incubated overnight at 4 °C. Species-specific HRP-conjugated secondary antibodies were used to label the primary antibodies. The Pierce ECL Western blotting substrate (Thermo Fisher Scientific, Waltham, MA, USA) was employed to develop the blots using the ChemiDoc™ Imaging System (Bio-rad, Hercules, CA, USA) and densitometry analysis was carried out using the Image lab software System 6.0.1 (Bio-rad, Hercules, CA, USA).

### 2.8. Statistical Analysis

Results for qRT-PCR analysis are presented as mean ± SEM. Results for immunohistochemistry, immunofluorescence, and Western blotting are presented as mean ± SD. One-way analysis of variance (ANOVA) with the Bonferroni–Dunn post hoc test was used to analyze and compare data between three or more groups. Data analysis was performed using Graphpad Prism software (version 10.1.0, San Diego, CA, USA), and *p* ≤ 0.05 was considered to be statistically significant.

## 3. Results

### 3.1. Prolonged Antibiotic Administration in GWI Mice Exacerbates Renal Inflammation and Production of Pro-Inflammatory Cytokines, Namely, IL-1β and IL-17A

**Figure 1:**
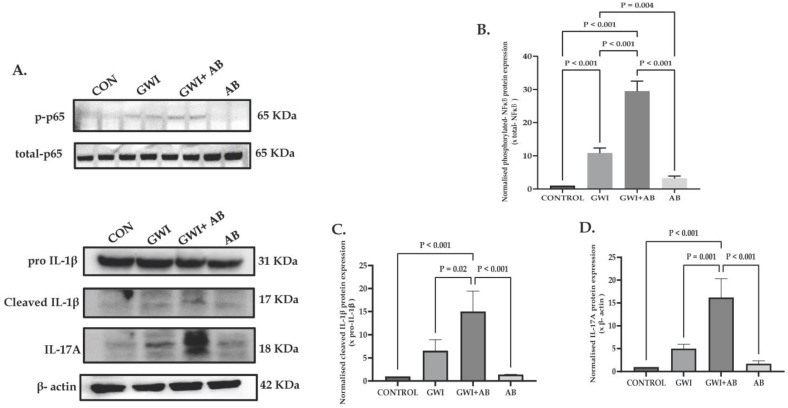
Analysis of Western blot data revealed that prolonged antibiotic administration (neomycin and enrofloxacin) in GWI mice resulted in increased kidney inflammation and elevated levels of pro-inflammatory cytokines, namely, IL-1β and IL-17A. Representative (**A**) depicts protein expression of phosphorylated-p65, total-p65, pro-IL-1β, cleaved IL-1 β, IL-17A, and β-actin in kidney tissue of CONTROL, GWI (persistent Gulf War Illness), GWI+AB (persistent Gulf War Illness with prolonged antibiotics exposure), and AB mice (prolonged antibiotics exposure only) (n = 6/group). (**B**–**D**) represents densitometry analysis of phosphorylated-p65 normalized with total-p65 (**B**), cleaved IL-1β normalized with pro-IL-1β (**C**), and IL-17A normalized with β-actin (**D**) in CONTROL, GWI, GWI+AB, and AB mice plotted as bar graph. The data are presented as the mean ±  SD (SD: standard deviation) and statistical significance was measured using one-way ANOVA between all the groups, with the Bonferroni–Dunn post hoc test. *p* < 0.05 was considered statistically significant.

To study the effect of prolonged antibiotic exposure in mice induced with GWI and its association with renal inflammation, we investigated the protein expression of NF-κB activation markers and pro-inflammatory cytokines such as IL-1β and IL-17A. The results indicated that the kidneys of GWI mice that were exposed to a prolonged antibiotic regime (GWI+AB) led to persistent NF-κB signaling activation marked by increased p65 phosphorylation (phosphorylated p65 expression normalized against Total-p65) when compared to GWI mice (Figure 1A,B, *p* < 0.001 between the GWI+ AB and GWI group). Mice treated with antibiotics only (AB) exhibited a marginal increase in p65 phosphorylation, although this increase was not statistically significant when compared to the control group (CONTROL). Additionally, when examining downstream targets of NF-κB signaling, we observed a notable significant increased protein expression of IL-1β (cleaved IL-1β expression against pro-IL-1β) and IL-17A (IL-17A expression normalized against β-actin) in the GWI+AB group compared to GWI mice (Figure 1A,C,D; for IL-1β- *p* = 0.02 and for IL-17A- *p* = 0.001 between the GWI+ AB and GWI group), suggesting that the exposure to antibiotics for a prolonged period might not be effective in mitigating the inflammatory response associated with GWI but plays a role in exacerbating renal inflammation.

### 3.2. Prolonged Antibiotic Administration in GWI Mice Increased in the Production and mRNA Expression of Pro-Inflammatory Cytokines and Chemokines

**Figure 2:**
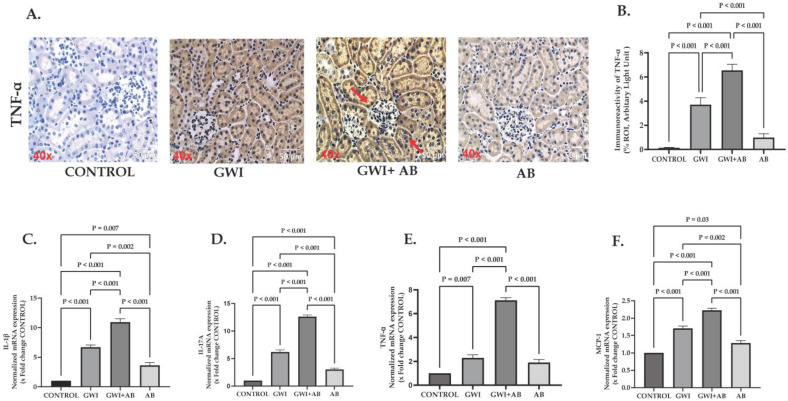
Immunohistochemical analysis and mRNA expression levels demonstrated that prolonged antibiotic administration (neomycin and enrofloxacin) in GWI mice led to an increase in the production of pro-inflammatory cytokines. Representative immunohistochemistry images (**A**) depict immunoreactivity of TNF-α in kidney sections from CONTROL, GWI (persistent Gulf War Illness), GWI+AB (persistent Gulf War Illness with prolonged antibiotics exposure), and AB mice (prolonged antibiotics exposure only) (n = 6/group). Images were captured at a 40× magnification (scale bar = 50 µm). Immunoreactivity was denoted by the presence of red arrows and morphometric analysis was calculated as %ROI. (**B**) The bar graph represents the morphometric analysis of TNF-α in CONTROL, GWI, GWI+AB, and AB mice. The data are presented as the mean ±  SD (SD: standard deviation) of %ROI (mean value calculated from five different fields in each sample). Bar graphs (**C**–**F**) illustrate the normalized mRNA expression levels of different pro-inflammatory cytokines, namely IL-1 β (**C**), IL-17A (**D**), and TNF-α (**E**), and chemokines, specifically MCP-1 (**F**) against GAPDH. These expression levels are presented as fold changes relative to the CONTROL derived from kidney tissue samples of CONTROL, GWI, GWI+AB, and AB mice. The experiment was performed in triplicates and the data are represented as mean ± SEM (SEM: standard error mean). Statistical significance was determined using one-way ANOVA with the Bonferroni–Dunn post hoc test. *p* < 0.05 was considered statistically significant.

To further evaluate the effect of prolonged administration of antibiotics resulting in the presence of persistent renal inflammation, the immunoreactivity of TNF-α and the genetic expression of IL-1β, IL-17A, TNF-α, and MCP-1 were studied. TNF-α is typically absent in healthy kidneys; however, upon stimulus, the TNF-α expression pattern in the kidneys appears to be associated with the specific compartment that is primarily affected [28]. In kidneys, TNF-α can originate from infiltrating inflammatory cells, podocytes, mesangial cells, and epithelial cells in proximal tubules and collecting ducts. TNF-α levels increase in response to stimuli such as endotoxemia, DAMPS or PAMPS-mediated receptor activation, oxidative stress, and complement activation [29,30]. In our study, we observed a significant increase in TNF-α immunoreactivity (marked by red arrows) in the renal tissue of GWI-treated mice compared to the control group (Figure 2A,B; *p* < 0.001). Notably, mice with GWI treatment co-administered with antibiotics for 20 weeks exhibited an even more substantial increase in TNF-α immunoreactivity in the renal tissue as compared to the GWI-treated mice (Figure 2A,B; *p* < 0.001). We also studied the genetic profile of various pro-inflammatory cytokines produced in the kidneys by qRT-PCR. Results indicate that the mRNA expression of IL-1β was significantly upregulated in GWI + AB mice as well as in GWI mice compared to the control (Figure 2C; *p* < 0.001) but the mRNA expression of IL-1β was more pronounced in the GWI+ AB group than in the GWI group (Figure 2C; *p* < 0.001). mRNA expression of IL-1β was also significantly increased in the kidneys of the AB group mice compared to the control but not to the extent of GWI or GWI+ AB mice (Figure 2C; *p* < 0.007 between the AB group and control). Similarly, the mRNA expression of IL-17A (Figure 2D), TNF-α (Figure 2E), and MCP-1 (Figure 2F) were also significantly upregulated in GWI+ AB mice compared to the GWI mice and control (for IL-17A, *p* < 0.001 between GWI+ AB and GWI, control, or AB group; for TNF-α, *p* < 0.001 for GWI+ AB group and GWI, control or AB group and *p* = 0.007 between GWI and control group; for MCP-1, *p* < 0.001 between GWI+ AB and GWI, control, or AB group) These results indicated that the administration of antibiotics in GWI mice led to a heightened inflammatory response in the kidneys compared to GWI mice, suggesting a complex interplay between GW chemicals and antibiotic exposure leading to increased renal inflammation.

### 3.3. Prolonged Antibiotic Administration Led to Augmentation of RAGE Activation Mediated by HMGB1 in GWI Mice 

**Figure 3:**
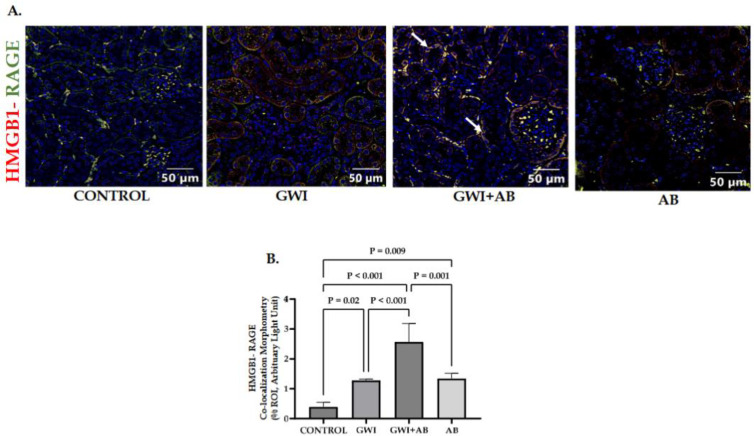
Immunofluorescence analysis demonstrated that prolonged antibiotic administration in GWI mice led to an augmentation in RAGE activation mediated by HMGB1. Representative (**A**) immunofluorescence images depict co-localization events (yellow) of HMGB1 (in red) and RAGE (in green) within kidney sections from CONTROL, GWI (persistent Gulf War Illness), GWI+AB (persistent Gulf War Illness with prolonged antibiotics exposure), and AB mice (prolonged antibiotics exposure only) (n = 6/group). The kidney sections were additionally counterstained with DAPI (in blue), and all images were acquired at a 40× magnification (scale bar = 50 µm). Immunoreactivity was denoted by the presence of white arrows and morphometric analysis was calculated as %ROI. (**B**) The bar graph represents HMGB1-RAGE co-localization events in CONTROL, GWI, GWI+AB, and AB mice. The data are presented as the mean ±  SD (SD: standard deviation) of %ROI (mean value calculated from five different fields in each sample) and statistical significance was measured using one-way ANOVA between all the groups, with the Bonferroni–Dunn post hoc test. *p* < 0.05 was considered statistically significant.

HMGB1 is a widely conserved nuclear protein that plays a pivotal role in numerous biological processes and is renowned for its ability to initiate immune responses as a damage-associated molecular pattern (DAMP) when found in the extracellular milieu. Previous research from our lab has documented elevated circulating levels of HMGB1, which have been associated with the development of gastrointestinal and neuro-inflammation in both sub-acute and persistent mouse models of GWI [31,32]. Increased concentrations of HMGB1 have been observed in both serum and urine among individuals with renal diseases. Various renal cell types such as podocytes, mesangial cells, tubular cells, endothelial cells, and lymphocytes express HMGB1; however, during injury, the primary source of HMGB1 expression is the renal tubular epithelial cells and podocytes [33]. HMGB1 triggers the release of pro-inflammatory cytokines and chemokines by interacting with TLRs and RAGE [30]. Following this rationale, we hypothesized the involvement of HMGB1-mediated RAGE activation led to the inflammatory phenotype in the kidneys. An immunofluorescence staining technique was performed to label kidney sections for studying the co-localization of the HMGB1 (red)/RAGE (green) interaction. We observed that the co-localization events (observed in yellow color) in the proximal convoluted tubular region of the kidneys (marked by white arrows) were significantly increased in the GWI+ AB group compared to the GWI group and control (Figure 3A,B; *p* < 0.001 between the GWI+ AB and GWI or control group). The GWI and AB group also showed a significant increase in co-localization events as compared to the control, but not to the extent of the GWI+ AB group (Figure 3A,B, *p* = 0.02 between the GWI and control; *p* = 0.009 between the AB and control group) indicating a potential role of HMGB1-mediated RAGE activation in the development of renal inflammation.

### 3.4. Prolonged Antibiotic Administration Is Associated with Worsened Renal Pathology by Increasing TGF-β Production in GWI Mice 

**Figure 4:**
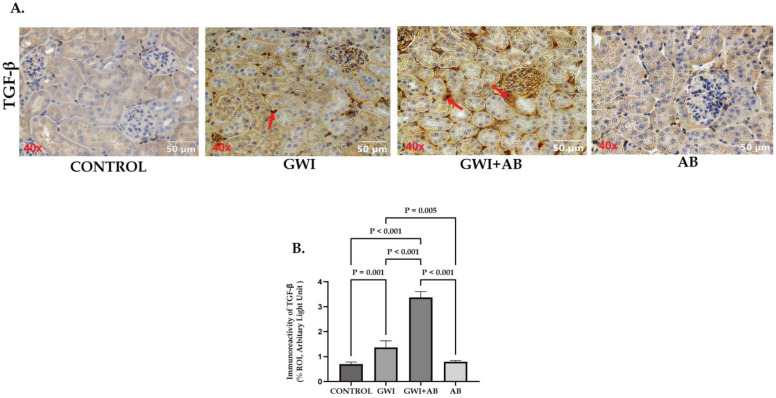
Immunohistochemical analysis demonstrated that prolonged antibiotic administration in GWI mice led to an increase in renal TGF-β production. Representative immunohistochemistry images (**A**) depict immunoreactivity of TGF-β in kidney sections from CONTROL, GWI (persistent Gulf War Illness), GWI+AB (persistent Gulf War Illness with prolonged antibiotics exposure), and AB mice (prolonged antibiotics exposure only) (n = 6/group). Images were captured at a 40× magnification (scale bar = 50 µm). Immunoreactivity was denoted by the presence of red arrows and morphometric analysis was calculated as %ROI. (**B**) The bar graph represents a morphometric analysis of TGF-β in CONTROL, GWI, GWI+AB, and AB mice. The data are presented as the mean ± SD (SD: standard deviation) of %ROI (mean value calculated from five different fields in each sample). Statistical significance was determined using one-way ANOVA with the Bonferroni–Dunn post hoc test. *p* < 0.05 was considered statistically significant.

Many studies have reported the potential role of TGF-β production in the pathogenesis of renal fibrosis because of its role in regulating cellular functions such as inflammation, differentiation, apoptosis, and proliferation [34,35]. Previous studies have reported the potential role of HMGB1-induced RAGE activation-mediated TGF-β secretion in PTECs leading to fibrosis-like pathology in various renal diseases such as diabetic nephropathy [36,37]. Thus, to investigate the role of prolonged antibiotic exposure in GWI mice leading to HMGB1-RAGE-mediated production of pro-fibrotic markers like TGF-β, we studied the immunoreactivity of TGF-β by immunohistochemistry in kidneys. Figure 4A highlights the immunoreactivity of TGF-β (marked by red arrows) in the interstitial space of the kidney section of control, GWI, GWI+ AB, and AB mice. Our results indicated that the kidney sections of GWI mice exposed to antibiotics for a prolonged period had significantly increased TGF-β production compared to GWI mice and control groups, respectively (Figure 4B; *p* < 0.001 between the GWI+ AB and GWI or control group). The kidneys of GWI mice also showed a significantly increased immunoreactivity of TGF-β secretion in comparison to control and AB groups; however, its production was subtle when compared to GWI+ AB mice (Figure 4B; *p* = 0.001 between the GWI and control, *p* = 0.005 between the GWI and AB group). This result highlighted that the prolonged antibiotic exposure in GWI mice worsened renal pathology by increasing the production of pro-fibrotic marker-TGF-β, potentially mediated through HMGB1-RAGE activation.

### 3.5. Prolonged Antibiotic Administration in GWI Mice Mediated the Upregulation of micro-RNA 21 (miR-21), a Signature miRNA for Renal Fibrosis That Targets the TGF-β/SMAD Pathway

**Figure 5:**
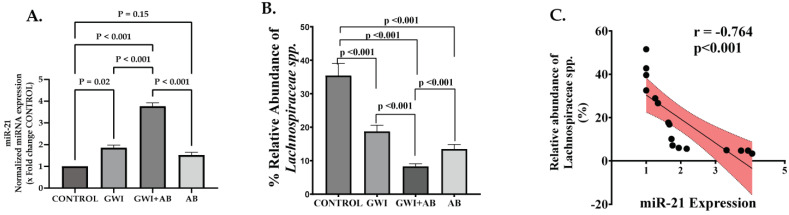
Prolonged antibiotic administration in GWI mice mediated the upregulation of micro-RNA 21 (miR-21), a signature miRNA for renal fibrosis, and correlated with *Lachnospiraceae* abundance. (**A**) represents miR-21 expression analyzed by qRT-PCR in the kidney tissue of CONTROL, GWI (persistent Gulf War Illness), GWI+AB (persistent Gulf War Illness with prolonged antibiotics exposure), and AB mice (prolonged antibiotics exposure only) (n = 6/group). The miR-21 expression was normalized using miR-103-3p as endogenous control. The bar graph is represented as fold change against CONTROL. (**B**) represents a bar graph showing the percent relative abundance of *Lachnospiraceae* spp. in CONTROL, GWI, GWI+AB, and -AB groups following whole genome sequencing of mouse fecal pellets. (**C**) depicts the correlation analysis of *Lachnospiraceae* spp. relative abundance and miR-21 levels in renal tissue of CONTROL, GWI, GWI+AB, and AB groups. Pearson’s linear regression is shown in red with 95% confidence bands. The experiment was performed in triplicates and the data are represented as mean ± SEM (SEM: standard error mean). Statistical significance was determined using one-way ANOVA with the Bonferroni–Dunn post hoc test. *p* < 0.05 was considered statistically significant.

One of the extensively studied miRNAs due to its role in various processes such as apoptosis, fibrosis, and cell proliferation is miR-21. The expression of miR-21 is upregulated in various kidney diseases such as renal fibrosis, CKD, acute kidney injury, and renal cancer. In healthy kidneys, miR-21 is predominantly found in the cortex, where its expression is maintained below a threshold with limited functionality. However, in the event of kidney injury, miR-21 expression increases, becoming prominent in the region of tubular epithelium within the kidneys. This pattern suggests that miR-21 primarily regulates genes located in the tubular epithelium when tissue damage occurs [38]. Additionally, TGF-β is reported to regulate the expression of several miRNAs including miR-21 upregulation, suggesting its role in TGF-β induced renal fibrosis through TGF-β/Smad3 signaling [39]. To investigate the potential relationship between increased TGF-β production and miR-21 expression due to the use of prolonged antibiotics in GWI mice, we investigated the expression profile of miR-21 in kidney tissues by qRT-PCR. We found that the expression of miR-21 was significantly increased in the kidney tissues of GWI+ AB mice compared to the GWI, control, or AB group (Figure 5A, *p* < 0.001 between GWI+ AB and GWI, control, or AB group). A significant but subtle increased miR-21 expression was also observed in the kidneys of the GWI group compared to the control group (Figure 5A, *p* = 0.02 between GWI and control). To find an association of gut dysbiosis in species abundance with miR-21 levels in the kidney tissue, we conducted a detailed microbiome analysis of our existing database from the same groups. Results showed that out of the 16 species that had differential abundance in the GWI+ AB group, only *Lachnospiraceae* had a significant negative correlation with miR-21 suggesting that a low abundance of *Lachnospiraceae* species caused by prolonged antibiotic administration has a statistically significant correlation with increased levels of miR-21 (Figure 5B,C, *p* < 0.001; *r =* −0.764). These results suggest that prolonged antibiotic exposure aggravates GW chemical exposure-induced miR-21 upregulation, a signature miRNA for renal fibrosis that may be associated with increased TGF-β production in the kidney microenvironment along with decreased relative abundance of *Lachnospiraceae* spp. observed in GWI+ AB mice.

### 3.6. Protein Expression of PTEN, a Target of miR-21, Is Perturbed Due to Prolonged Antibiotic Treatment in GWI Mice, Thereby Modulating AKT Signaling, as PTEN Functions as a Negative Regulator of AKT Signaling

miR-21 regulates the expression of its target genes via post-transcriptional mechanisms. miRNAs are transcribed by RNA Polymerase II, much like mRNAs, and within the nucleus, primary miRNA transcripts are processed by Dorsha RNase III giving rise to stem-loop-like structures that contain pre-miR. These pre-miR are transported to the cytoplasm where they undergo further processing by various proteins leading to the formation of 22 nucleotide long double-stranded RNAs from which the miRNA guide strand is generated. This miRNA guide strand interacts within the RISC complex and engages with specific miRNA recognition elements at the 3′-UTR of the target mRNAs. This binding between miRNA-mRNA results in suppression of mRNA translation, which might also result in mRNA degradation [40]. PTEN and SMAD7 are reported to be the direct targets of miR-21. miR-21 plays an important role by targeting PTEN and Smad7 in the progression of renal fibrosis. Further, PTEN acts as a negative regulator of AKT signaling, thereby contributing to cellular inflammation, proliferation, survival, glucose metabolism, and other processes.

**Figure 6:**
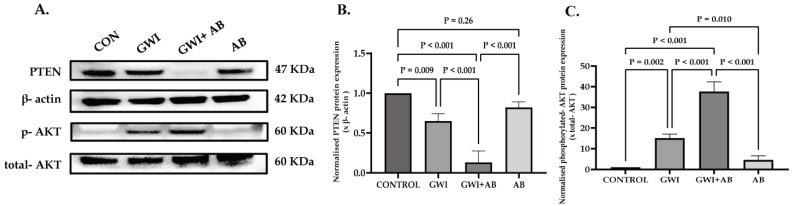
The protein expression of PTEN, a target of miR-21, is perturbed due to prolonged antibiotic treatment in GWI mice, thereby modulating AKT signaling, as PTEN functions as a negative regulator of AKT signaling. (**A**) represents Western blot analysis of the relative protein expression of PTEN, phosphorylated-AKT, total-AKT, and β-actin in kidney tissue of CONTROL, GWI (persistent Gulf War Illness), GWI+AB (persistent Gulf War Illness with prolonged antibiotics exposure), and AB mice (prolonged antibiotics exposure only) (n = 6/group). (**B**,**C**) represents a bar graph illustrating the densitometry analysis of PTEN normalized with β-actin (**B**) and phosphorylated-AKT normalized with total-AKT (**C**) in CONTROL, GWI, GWI+AB, and AB mice plotted as a bar graph. The data are presented as the mean ± SD (SD: standard deviation) and statistical significance was measured using one-way ANOVA between all the groups, with the Bonferroni–Dunn post hoc test. *p* < 0.05 was considered statistically significant.

To explore this mechanism, we assessed the protein expression of PTEN in kidney tissues of control, GWI, GWI+ AB, and AB mice by Western blotting. Figure 6A. shows the representative immunoblots of PTEN, β-actin, phosphorylated AKT, and total-AKT in the kidney tissues of control, GWI, GWI+ AB, and AB mice. We observed that the protein expression of PTEN decreased significantly in the GWI+ AB group compared to the GWI, control, and AB group, indicating that increased miR-21 expression in the renal tissue of GWI+ AB mice interferes with PTEN gene expression through post-transcriptional repression of its mRNA. Kidneys of GWI mice also showed a marginal decreased expression of PTEN compared to the control and AB group (Figure 6A,B, *p* = 0.009 between GWI and control group). However, expression of PTEN was significantly higher in the GWI group than in the GWI+ AB group, suggesting a potential association between prolonged antibiotic exposure and increased TGF-β-induced miR-21 expression, leading to the downregulation of PTEN in renal tissues (Figure 6A,B, *p* < 0.001 between the GWI+ AB and GWI group). Further, to validate whether TGF-β-induced miR-21 expression results in AKT signaling activation via PTEN, we studied the phosphorylation status of AKT protein at the serine-473 position. Our results showed that activation of AKT signaling was significantly increased in the renal tissue of GWI mice compared to control or AB mice (Figure 6A,C, *p* = 0.002 between GWI and control; non-significant (ns) between the GWI and AB group). However, a significantly increased AKT phosphorylation at the Ser-473 position, indicating intense activation of AKT signaling, was observed in the renal tissue of GWI+ AB mice than in GWI mice (Figure 6A,C, *p* < 0.001 between the GWI+AB and GWI group). These results suggested that the use of antibiotics for long periods exacerbated kidney inflammation and fibrosis-like pathology by further increasing activation of AKT signaling through miR-21-induced PTEN downregulation.

### 3.7. Prolonged Antibiotic Treatment in GWI Mice Caused Increased Extracellular Matrix (ECM) Deposition and the Induction of an Epithelial–Mesenchymal Transition (EMT)-like Phenotype in the Kidney

**Figure 7:**
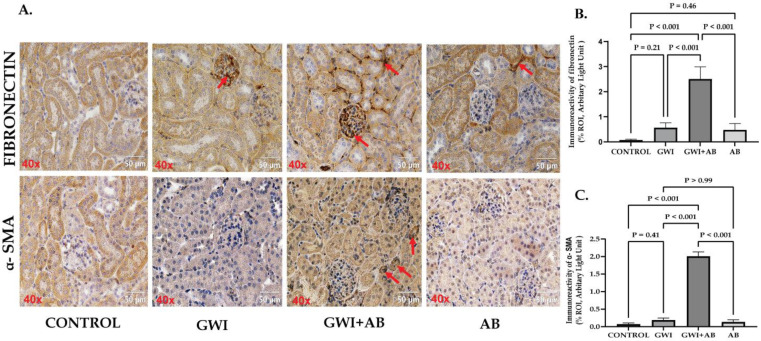
Prolonged antibiotic treatment in GWI mice triggers the activation of AKT signaling, resulting in increased extracellular matrix deposition (ECM) and the induction of epithelial–mesenchymal transition (EMT)-like phenotype. Representative immunohistochemistry images (**A**) depict immunoreactivity of Fibronectin and α-SMA in kidney sections from CONTROL, GWI (persistent Gulf War Illness), GWI+AB (persistent Gulf War Illness with prolonged antibiotics exposure), and AB mice (prolonged antibiotics exposure only) (n = 6/group). Images were captured at a 40× magnification (scale bar = 50 µm). Immunoreactivity was denoted by the presence of red arrows and morphometric analysis was calculated as %ROI. (**B**,**C**) represents a bar graph illustrating the morphometric analysis of Fibronectin (Figure 6B) and α-SMA (Figure. 6C) in CONTROL, GWI, GWI+AB, and AB mice. The data are presented as the mean ±  SD (SD: standard deviation) of %ROI (mean value calculated from five different fields in each sample). Statistical significance was determined using one-way ANOVA with the Bonferroni–Dunn post hoc test. *p* < 0.05 was considered statistically significant.

Renal fibrosis, characterized by marked increased ECM deposition and activation of EMT is a hallmark of most of the CKD. Studies have reported that the activation of AKT signaling by miR-21 targeted PTEN downregulation leads to phenotypic alterations in tubular epithelial cells aggravating renal fibrosis [25,41,42]. Thus, to investigate the role of AKT signaling activation on renal fibrosis, we performed immunohistochemistry to evaluate the markers-fibronectin for ECM deposition and α-SMA for EMT initiation. Immunohistochemistry data revealed a non-significant increase in fibronectin deposition in the renal tissue of GWI mice compared to control or AB mice (Figure 7A,B, *p* = 0.21 between the GWI and control group). Compared to GWI mice, the GWI+ AB mice showed a significantly increased fibronectin deposition in the renal interstitial space (Figure 7A,B, *p* < 0.001 between the GWI + AB and GWI group). α-SMA serves as a marker for the cells that have undergone EMT, and its expression in epithelial cells is indicative of their transition to a mesenchymal state. We observed significant immunoreactivity of α-SMA in the renal tissue of GWI+ AB mice only (Figure 7A,C, *p* < 0.001 between the GWI+ AB and control, GWI, and AB group), whereas no significant α-SMA immunoreactivity was observed in GWI mice (Figure 7A,C, *p* = 0.41 between the GWI and control group; *p* > 0.99 between the GWI and AB group) indicating that prolonged exposure to antibiotics amplifies the effects of GW chemicals, resulting in the development of a pathology resembling renal fibrosis. This pathology is marked by increased expression of α-SMA and deposition of fibronectin.

## 4. Discussion

Studies have reported that prolonged use of certain classes of antibiotics such as fluoroquinolones, aminoglycosides, β-lactams, and glycopeptides in diseased conditions may exert detrimental effects, some of which are strongly associated with various renal diseases such as interstitial nephropathy, tubular cell toxicity, and tubulointerstitial fibrosis [8,43,44]. Also, it has been well speculated that misuse or overuse of such antibiotics might compromise the body’s immune system to fight against diseases and may contribute to further exaggerated complications [45]. Interestingly, a study reported that Aminoglycosides (neomycin) and fluoroquinolone (enrofloxacin) classes of antibiotics were among the most used antibiotics in veterans hospitalized with pneumonia [46]. However, we are not aware of any studies that report the frequency or detailed record of long-term antibiotic use in veterans. Therefore, this study was undertaken to investigate the risks associated with the combined effects of long-term antibiotic exposure, particularly of class fluoroquinolone–enrofloxacin and aminoglycosides–neomycin with GW chemicals in persistent mouse models on renal pathology. We showed a novel preclinical assessment of renal fibrotic pathology risk following the prolonged use of antibiotics, a possible clinical scenario that is prevalent amongst veterans with GWI. Further, the large burden of comorbidities in US veterans with newly identified chronic kidney disease places them at the risk of premature death [47]. 

Chronic renal inflammation is characterized as the initial reaction of the kidneys in response to stress or injury, leading to the progression of renal fibrosis [48]. Activation of NFκB signaling has been reported in podocytes, mesangial cells, and tubular epithelial cells in various renal diseases such as glomerular injury, tubulointerstitial diseases, and acute kidney injury (AKI) [49,50,51]. Activation of NF-κB signaling plays a significant role in chronic inflammation associated with chronic kidney diseases. Initially, NF-κB is inactive and located in the cytoplasm, where it is restrained by IκB proteins. Upon activation, IκB kinase phosphorylates and degrades IκB proteins, allowing NF-κB to move into the nucleus. This activation of NF-κB is marked by the phosphorylation of various subunits, with common combinations being p50/NF-κB1-p65/RelA and p50/NF-κB1-c-Rel, leading to the transcription of pro-inflammatory genes [52]. In this study, we observed that prolonged antibiotic administration in GWI mice exacerbated renal inflammation by significantly increasing NFκB signaling activation with increased protein and mRNA expression of various pro-inflammatory cytokines-IL-1β, IL-17A, and chemokines-MCP-1. In the kidneys of GWI mice, inflammation was evident, accompanied by elevated levels of pro-inflammatory cytokines, albeit to a lesser degree. Further, we also observed increased TNF-α immunoreactivity in the renal tissue and increased mRNA expression of MCP-1 in the kidney tissue of GWI+ AB mice compared to GWI mice. MCP-1 is known to be upregulated during inflammation and NFκB activation [53]. Moreover, elevated TNF-α is also reported to stimulate the release of MCP-1, thereby playing a major role in renal inflammation and fibrosis [54]. It has been reported that activation of tubular epithelial cells by MCP-1 in vitro contributes to tubulointerstitial inflammation, a hallmark of progressive renal disease [55].

Binding of pathogen-associated molecular patterns (PAMPs) and/or damage-associated molecular patterns (DAMPs) by pattern-recognition receptors (PRRs) located on both innate immune cells and epithelial cells triggers the onset of inflammation [56]. HMGB1 is a nuclear protein that is expressed by many renal cell types including tubular epithelial cells. In the event of tissue injury, HMGB1 translocates from the nucleus to the cytoplasm and is released into the extracellular space [57]. In the extracellular space, HMGB1 binds to specific cell surface receptors such as TLRs (TLR2, TLR4) and RAGE to exert its effects. Previously, our lab and others have reported increased HMGB1 levels in the serum of a persistent GWI mouse model, and that GW veterans are associated with neuroinflammation via HMGB1-RAGE activation-mediated blood–brain barrier dysfunction [31]. Many reports have identified the extensive role of RAGE activation in renal inflammation and fibrosis. Studies have shown that HMGB1-mediated RAGE activation results in the production of connective tissue growth factor (CTGF) and TGF-β, thereby promoting renal fibrosis in tubular epithelial cells [11,36]. Because of the versatile role of HMGB1, we decided to explore the role of HMGB1-RAGE activation in renal inflammation. Here, we observed a significant marked increase in events of HMGB1-RAGE co-localization in the renal tissue of GWI mice exposed to prolonged antibiotic treatment. We also observed increased HMGB1-RAGE activation in GWI mice; however, the activation was less prominent than in the GWI+ AB mice. These data suggest that the use of antibiotics for extended periods intensifies the effect of GW chemicals by increasing HMGB1 release into the extracellular environment. HMGB1 might interact with RAGE or other cell surface receptors and mediate renal inflammation by increasing the production of pro-inflammatory cytokines.

TGF-β is considered a key profibrotic mediator in various chronic kidney diseases. Both canonical and non-canonical TGF-β/Smad signaling plays a significant role in renal fibrosis [58]. MicroRNAs are single-stranded, small, non-coding RNAs 20–22 nucleotides long that modulate a variety of biological functions including inflammation and fibrosis. Upregulation of miR-21 has been extensively observed in various renal diseases including renal fibrosis [59,60,61]. Recent evidence has claimed that TGF-β induces interaction between phosphorylated-Smad3 and the miR-21 promoter which upregulates miR-21 expression, indicating a potential role of TGF-β/Smad3 signaling in miR-21 transcription [39]. In our study, we found increased TGF-β production in the GWI mice kidneys that were exposed to prolonged antibiotics compared to the renal tissue of GWI mice. However, the upregulation of miR-21 was more significantly pronounced in GWI+ AB mice kidneys compared to GWI mice. These results were found to be consistent with other findings that have reported the correlation of upregulated miR-21 expression with increased TGF-β production in tubular epithelial cells of mice kidneys [22,27,62]. These studies also validated their findings using both human and rat tubular epithelial cells such as HK-2, HKC, and NRK-52E cell lines. However, a noteworthy limitation of our study is the challenge of replicating our intended extended antibiotic treatment in an in vitro setting. Further, though we have found an increased TGF-β level in the renal tissue, we could not validate the exact role of TGF-β in inducing the miR-21 to claim the former’s direct role in the process. Another constraint in our study is the absence of miR-21 knockout mice, which could have served as a valuable tool for validating our findings in an in vivo context. Utilizing miR-21 knockout mice would have enhanced our ability to confirm and strengthen the relevance of our observations regarding the impact of antibiotic exposure in GWI mice.

PTEN and Smad7 are reported to be the direct targets of miR-21 [63]. PTEN functions as a tumor suppressor gene with antifibrotic effects. MiR-21 downregulates PTEN expression by binding to its mRNA which inhibits translation and promotes PTEN mRNA degradation. Decreased PTEN expression is associated with the progression of liver, lung, and kidney fibrosis, and the extent of organ fibrosis is positively correlated with the degree of PTEN downregulation [64,65]. This connection is further evidenced by studies like the one showing exacerbated renal collagen production, ECM deposition, and myofibroblast differentiation in Angiotensin II (AngII)-induced hypertension in mice with myeloid PTEN deficiency. Additionally, this study revealed increased F4/80+ macrophage infiltration and CD3+ T cell presence in the kidneys of these mice, highlighting PTEN’s crucial role in regulating renal inflammation and fibrosis [66]. Protein expression of PTEN was significantly downregulated in the kidney tissue of GWI mice exposed to antibiotics for five months than in GWI mice. Furthermore, PTEN also serves as a negative regulator of AKT activation [26]. Therefore, we evaluated AKT protein expression to delineate the effect of PTEN downregulation on AKT activation due to prolonged antibiotic treatment. Here, we found that the kidneys of GWI mice exposed to prolonged antibiotics showed increased AKT phosphorylation as compared to GWI-only mice. This indicated that subjecting GWI mice to a 5-month antibiotic exposure worsens renal pathology by intensifying TGF-β production which, in turn, leads to severe miR-21 upregulation, leading to heightened activation of the PTEN/AKT signaling pathway.

Renal fibrosis is reported to be the end point of progressive CKD, characterized by increased deposition of ECM proteins such as fibronectin, and collagen in both the tubulointerstitial and/or glomeruli region of the kidneys, but also includes the accumulation of activated fibroblasts and enhanced EMT [67]. Fibronectin is an essential high molecular-weight adhesive glycoprotein that is critical for wound healing and ECM formation. Expression of this glycoprotein increases fibrosis or post-tissue injury in various renal compartments such as the glomerular mesangium, Bowman’s capsule, and tubulo-interstitium [68]. The existing literature outlines multiple mechanisms through which miR-21 could contribute to the development of renal fibrosis. miR-21 upregulation results in increased M2 polarization of macrophages, which promotes fibrosis by secreting TGF-β1, which stimulates myofibroblasts activation, proliferation, and ECM deposition [69]. Another mechanism includes the involvement of STAT6, a Jumonji domain-containing protein-3 (JMJD3)-mediated increased M2 polarization as a critical regulator of myeloid fibroblast activation and renal fibrosis development [70,71,72]. This emphasizes the significance of M2 macrophage polarization in the progression of renal fibrosis. However, the role of macrophage infiltration in renal fibrosis progression is not addressed in our current study. Investigating the impact of miR-21 through M2 macrophage polarization could offer additional significant insights into the underlying molecular mechanisms for renal fibrosis associated CKD. Lan A et al. described that activation of AKT signaling regulates renal fibrosis by inducing EMT phenotype by increasing α-SMA expression and ECM deposition [73]. We observed that the renal sections of GWI mice exposed to prolonged antibiotics showed increased deposition of fibronectin and α-SMA expression. Contrarily, the renal sections of GWI mice did not show any significant deposition and expression of these markers. These data demonstrated that it is the prolonged administration of antibiotics that amplifies the renal insult caused by GW chemical exposure that leads to the development of renal fibrosis-like pathology in GWI mice. 

Interestingly, gut dysbiosis has been shown to be causally linked to chronic kidney disease and the abundance of host species plays a significant role. *Lachnospiraceae* spp. are major producers of short-chain fatty acids (SCFAs) by hydrolyzing starches and sugars and functions to promote good health. The prominent characteristic of dysbiosis in CKD is dysregulated levels of *Lachnospiraceae* spp. Further studies in UUO mice suggested distinct gut microbial composition at various stages of renal injury. A significant decrease in SCFAs producing *Bacteroides*, *Prevotellaceae_UCG-001*, *Roseburia*, and *Lachnospiraceae_NK4A136*_group in UUO-induced renal fibrosis mice suggests that alterations in the gut microbiome, particularly the decrease in SCFA-producing genera like Lachnospiraceae, may contribute to the progression of renal fibrosis [74]. It has also been recently shown that *Alcaligenaceae*, *Lachnospiraceae,* and *Ruminococcus torques* groups had a tendency to causally decrease the risk of CKD [75]. Lower levels of *Lachnospiraceae* are linked to diseases such as splenomegaly, lymphadenopathy, glomerulonephritis, and other renal diseases [76]. Our finding of a strong correlation between low *Lachnospiraceae* abundance in GWI+ AB mice and higher miR-21 levels and kidney pathology provides a further mechanistic understanding of the risk of CKD in GWI veterans.

Overall, these results highlight that exposure to GW chemicals alone triggers renal inflammation by activating HMGB1-RAGE-mediated production of pro-inflammatory cytokines and TGF-β production. We also showed increased mir-21 expression and downregulation of its target gene-PTEN in the kidney tissue of GWI mice followed by activation of AKT signaling. However, administration of antibiotics for 20 weeks worsens the condition of GWI mice exhibiting exacerbated inflammation along with increased TGF-β-mediated activation of miR-21/PTEN/AKT signaling. Further, no significant deposition of fibronectin or expression of EMT marker α-SMA was observed in the renal tissue of GWI mice. Contrastingly, the renal tissue of GWI mice co-exposed with antibiotic treatment for 20 weeks showed a significantly increased deposition of fibronectin and increased α-SMA immunoreactivity, indicating that the exposure to prolonged antibiotic exposure aggravates renal pathology induced by GW chemicals, further aiding in the development of renal fibrosis-like pathology, possibly via TGF-β-mediated miR-21/PTEN/AKT signaling. We aim to connect the above-described mediators by extending our studies by using knockout mouse models where both TGF-β and miR-21 are absent to confirm our reported findings.

## Figures and Tables

**Table 1:** Mouse primers sequence.

Gene	Primer Sequence (5′–3′)		Melting Temperature (Tm)
	Forward Sequence	Reverse Sequence	
GAPDH	CGACTTCAACAGCAACTCCCACTCTTCC	TGGGTGGTCCAGGGTTTCTTACTCCTT	62 °C
IL-1β	CCTCGGCCAAGACAGGTCGC	TGCCCATCAGAGGCAAGGAGGA	59.2 °C
IL-17A	TGAGCTTCCCAGATCACAGA	TCCAGAAGGCCCTCAGACTA	52.8 °C
TNF-α	CGTCAGCCCGATTTGCTATCT	CGGACTCCGCAAAGTCAAG	52.8 °C
MCP	CACAGTTGCCGGCTGGAGCAT	GTAGCAGCAGGTGAGTGGGGC	59.2 °C

## Data Availability

The data presented in this study are available upon request from the corresponding author.
